# Chamber‐Specific Morphometry and Quantitative Cardiac Histology in the Broad‐Snouted Caiman (*Caiman latirostris*)

**DOI:** 10.1111/ahe.70183

**Published:** 2026-10-16

**Authors:** Maria Eduarda Natal Nascimento, Gabriela Nogueira Maggio, Maysa Barbosa de Almeida, Caio Rafael Siqueira Vasconcelos, Vinícius José da Silva Cardoso de Brito, Arianne Pontes Oriá, Fernanda Gosuen Gonçalves Dias, Marcela Aldrovani Rodrigues

**Affiliations:** ^1^ Universidade de Franca (UNIFRAN) Franca São Paulo Brazil; ^2^ Universidade Federal da Bahia (UFBA) Salvador Bahia Brazil

**Keywords:** alligators and crocodiles, cardiomyocytes, heart, histology, stereology

## Abstract

Quantitative cardiac morphology remains poorly documented in crocodilians. Hearts from four commercially reared male *Caiman latirostris* were examined by gross morphometry, histology, stereology, reticulin staining and Masson's trichrome. Relative heart mass averaged 0.125% of body mass. The left ventricular wall was numerically thicker than the right. Cardiomyocyte volume density and cross‐sectional area varied among chambers, whereas collagen‐positive area was greater in the right ventricle. Pulmonary arterial walls were markedly thinner than the systemic aortae, and the left pulmonary arterial wall was thicker than the right. These findings extend quantitative cardiac reference data for this species.

## 1 Introduction

The crocodilian cardiovascular system combines complete atrial and ventricular septation with paired systemic aortae linked by the foramen of Panizza. This organisation separates pulmonary and systemic pressures while allowing state‐dependent redistribution of blood flow (Jones and Shelton 1993; Axelsson and Franklin 2001; Cook et al. 2017; Poulsen et al. 2024).

Macroscopic cardiac anatomy has been described in several crocodilian species, including *Caiman yacare* , *Alligator sinensis* and *C. latirostris* (Alves et al. 2016; de Oliveira Lima et al. 2021; Xie et al. 2023). In *C. latirostris* , the available study established cardiac topography and gross morphometry but did not quantify chamber‐specific cardiomyocyte organisation (de Oliveira Lima et al. 2021). It therefore remains unclear whether right–left differences in chamber dimensions are accompanied by tissue‐level differences. Stereological and morphometric approaches can address this gap by providing objective estimates of cardiomyocyte volume density and cell size (Mühlfeld et al. 2010; Mühlfeld and Schipke 2022).

This study therefore quantified cardiac morphology in commercially reared male *C. latirostris* by evaluating relative heart mass, chamber dimensions, total right and left ventricular wall thickness, cardiomyocyte volume density and cross‐sectional area, ventricular collagen‐positive area and wall thickness of the paired systemic aortae and right and left pulmonary arteries.

## 2 Materials and Methods

Heart specimens were obtained from four commercially reared male *C. latirostris* (body mass 10.8–23.4 kg) following routine commercial slaughter for meat and leather; slaughter was unrelated to the study. The animals originated from Mister Cayman Ltda. (Maceió, Alagoas, Brazil), where they were maintained under a closed‐cycle farming system, and were slaughtered at a state‐inspected facility operating under the Alagoas State Inspection Service (SIE/ADEAL). The farming facility was registered with the Brazilian Institute of Environment and Renewable Natural Resources (IBAMA registration no. 546538), and specimen collection was authorised by SISBIO/ICMBio (no. 27489‐8). Hearts were removed after slaughter, weighed, fixed for 48 h in 10% neutral‐buffered formalin and transported to the Laboratory of Animal Science, Universidade de Franca. Relative heart mass was calculated for each animal as heart mass divided by body mass and expressed as g kg −1 and as a percentage of body mass. After fixation, the hearts were rinsed in running water for 4 h before anatomical and biometric examination.

Cardiac position and relationships with adjacent structures were recorded before removal. External and internal morphology was then examined after dissection. Base‐to‐apex length, base, apex and lateral widths, and right and left atrial and ventricular widths were measured three times per heart with a digital calliper (CD‐12 CP, Mitutoyo Sul Americana, Suzano, Brazil). Total ventricular wall thickness was measured at three equidistant points along the free wall of each ventricle, at the mid‐ventricular level, perpendicular to the epicardial surface. Measurements extended from the epicardial surface to the deepest endocardial margin of the trabecular myocardium, thus encompassing both the compact and trabecular components. The mean of the three measurements represented each ventricle in each animal. Anatomical terminology followed the Nomina Anatomica Veterinaria.

Samples from the right and left atria, right and left ventricles, parietal pericardium (pericardial sac), cardiac valves, chordae tendineae, paired systemic aortae and right and left pulmonary arteries were processed for paraffin embedding. Longitudinal and transverse sections (3 μm) were stained with haematoxylin and eosin. Reticulin staining was used to demonstrate the myocardial argyrophilic reticular fibre network (Schwint et al. 2004). Sections of the parietal pericardium and ventricular myocardium were also stained with Masson's trichrome to demonstrate collagenous connective tissue. Sections were evaluated by bright‐field microscopy (Eclipse E200, Nikon, Tokyo, Japan), and images were acquired using 10× and 40× objectives.

Digital morphometry was performed on calibrated images using ImageJ (Schneider et al. 2012). Six non‐overlapping microscopic fields from each cardiac chamber of each animal were evaluated for cardiomyocyte volume density (Vv) and cross‐sectional area. Vv was estimated in STEPanizer (Tschanz et al. 2011) by superimposing a 36‐point grid and calculating ΣP(cardiomyocytes)/ΣP(total), expressed as a percentage. Cardiomyocyte cross‐sectional area was expressed in μm^2^. In Masson's trichrome‐stained sections, collagen‐positive area was quantified in five non‐overlapping fields from each ventricle of each animal and expressed as the percentage of blue‐stained tissue area after exclusion of unstained background. Total wall thickness of the systemic aortae and pulmonary arteries was measured in μm. Three equidistant wall measurements were obtained from each right and left pulmonary artery in each of the four hearts.

Descriptive statistics comprised the mean, standard deviation, median, and range. Normality was assessed with the Shapiro–Wilk test. Paired *t*‐tests were used for right–left comparisons of atrial and ventricular widths, total ventricular wall thickness, cardiomyocyte measurements, collagen‐positive area, and systemic aortic and pulmonary arterial wall thickness (*n* = 4 animals). Statistical significance was set at *p* < 0.05. Analyses were performed in GraphPad Prism 6.0 (GraphPad Software, San Diego, CA, USA).

## 3 Results

The heart occupied a central position in the cranial coelomic cavity, between the fourth and fifth intercostal spaces, and was enclosed by the pericardium. Its apex was directed slightly to the left. Each heart comprised two atria and two completely separated ventricles. The right systemic aorta, left systemic aorta and pulmonary trunk arose separately. The foramen of Panizza, atrioventricular valves, chordae tendineae, pectinate muscles, interventricular septum and compact and trabecular ventricular myocardium were readily identified (Figure 1).

**FIGURE 1:**
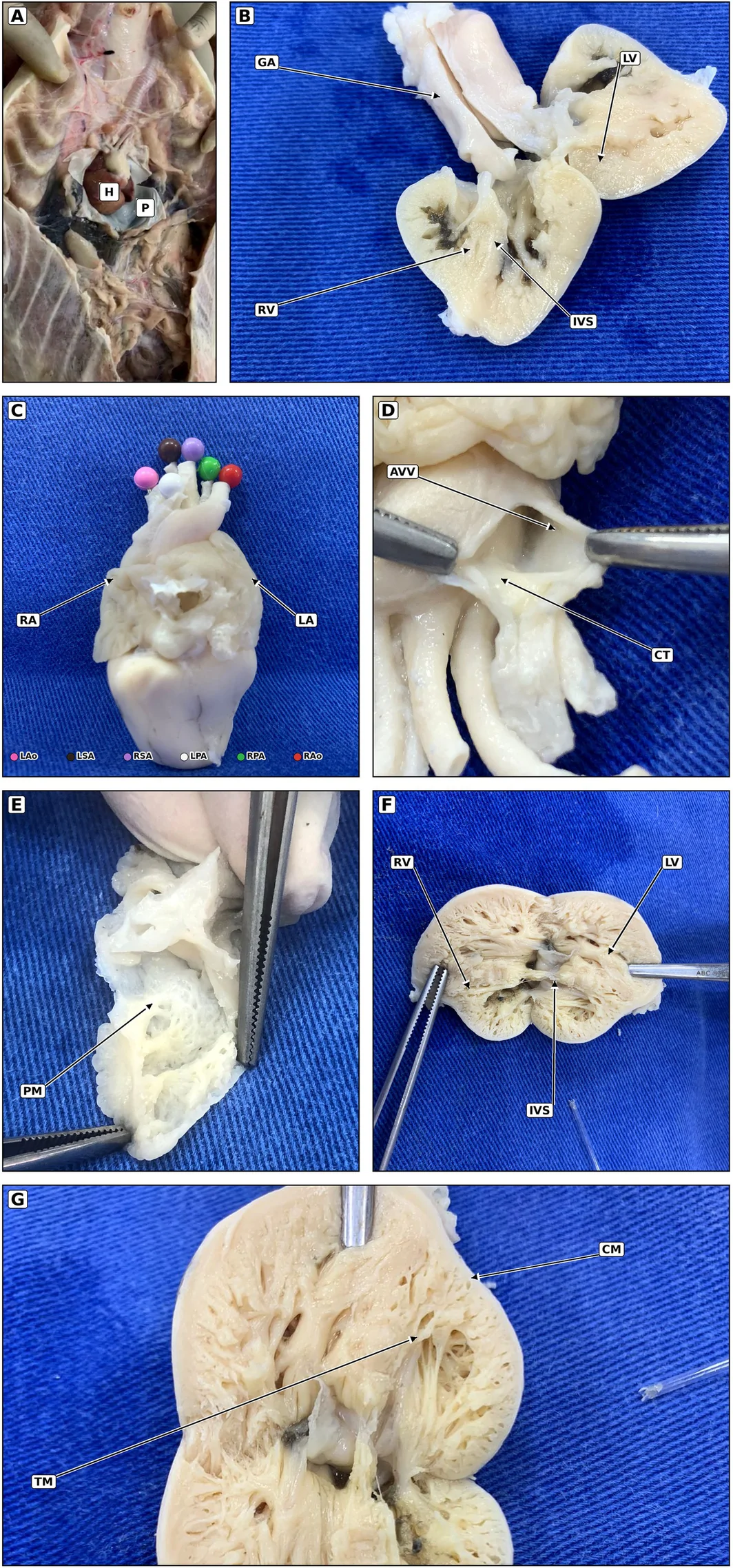
Macroscopic cardiac anatomy of *Caiman latirostris* . (A) In situ view of the cranial coelomic cavity showing the heart (H) enclosed by the parietal pericardium (P). (B) Dissected heart showing the right ventricle (RV), left ventricle (LV), interventricular septum (IVS), and great arteries (GA). (C) External view showing the right (RA) and left (LA) atria and the great arterial vessels identified with coloured pins: Right systemic aorta (RAo, red), left systemic aorta (LAo, pink), right pulmonary artery (RPA, green), left pulmonary artery (LPA, white), right subclavian artery (RSA, purple), and left subclavian artery (LSA, black). (D) Higher‐magnification view of an atrioventricular valve (AVV) and chordae tendineae (CT). (E) Interior of an atrium showing pectinate muscles (PM). (F) Opened ventricular chambers showing the RV, LV, and IVS. (G) Higher‐magnification view of the left ventricular wall showing compact myocardium (CM) and trabecular myocardium (TM).

Heart mass ranged from 15.42 to 26.67 g (mean 20.64 ± 5.42 g), and relative heart mass averaged 1.25 ± 0.19 g kg −1, equivalent to 0.125 ± 0.019% of body mass (range 0.101–0.143%; Figure 2D). Mean base‐to‐apex length was 45.84 mm, and the remaining macroscopic measurements are summarised in Table 1. The right atrium was wider than the left atrium, whereas the left ventricle was wider than the right ventricle (both *p* < 0.05; Figure 2A,B). Mean total ventricular wall thickness was 4.42 ± 1.41 mm in the right ventricle and 6.02 ± 1.64 mm in the left ventricle; this difference did not reach statistical significance (*p* = 0.075; Figure 2C).

**FIGURE 2:**
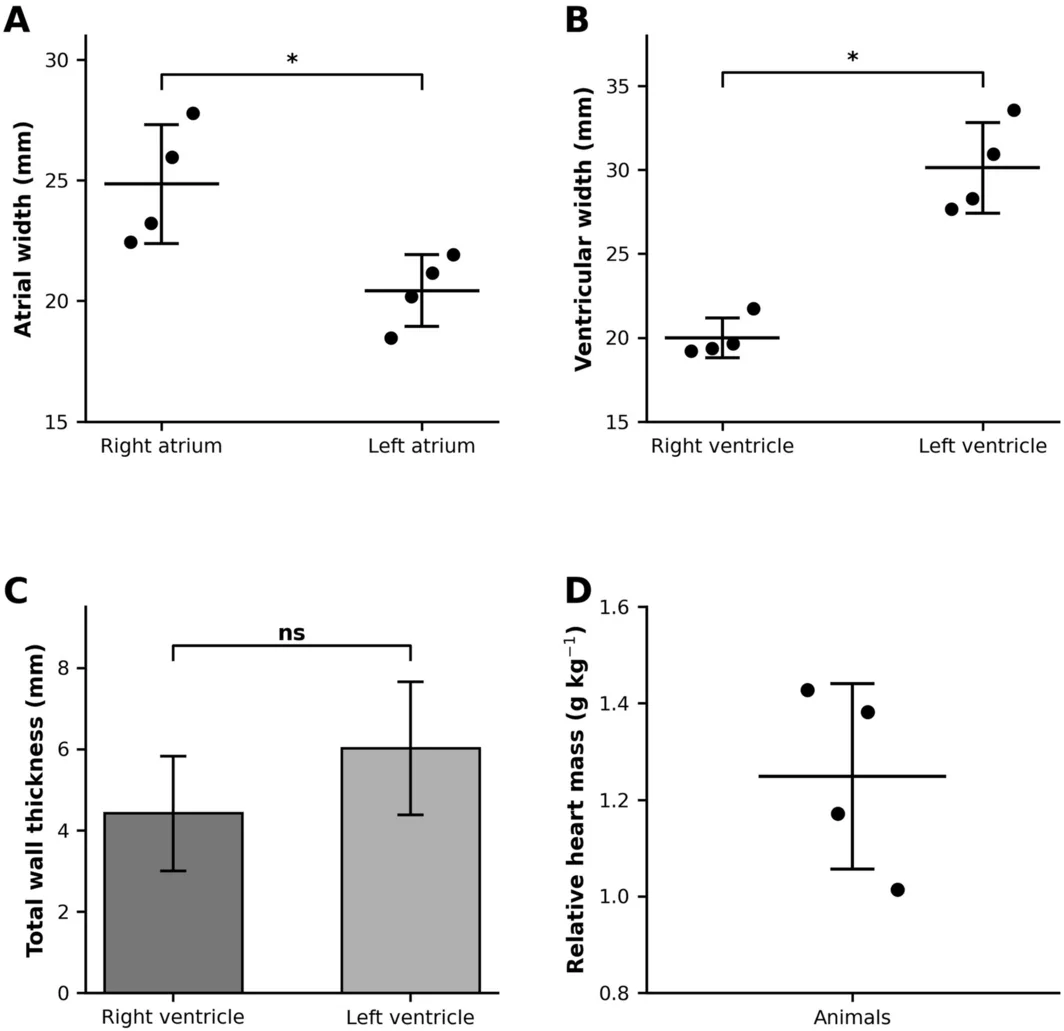
Gross cardiac morphometry of *Caiman latirostris* . (A) Right and left atrial widths; (B) right and left ventricular widths; (C) total right and left ventricular wall thickness, encompassing the compact and trabecular components; and (D) relative heart mass. Data were obtained from four animals. In A, B, and D, each symbol represents one animal and the central horizontal bars indicate the mean. In C, bars represent the mean. Error bars show standard deviation. Atrial and ventricular widths: *p < 0.05; ventricular wall thickness: *p* = 0.075.

**TABLE 1:** Macroscopic cardiac measurements in four commercially reared male *Caiman latirostris* (mm).

Parameter	Mean	SD	Median	Minimum	Maximum
Base‐to‐apex length	45.84	3.14	45.47	42.45	49.98
Base width	33.14	4.63	32.73	28.77	38.33
Apex width	13.49	2.49	12.59	11.67	17.13
Lateral width	28.69	5.37	29.22	21.76	34.56
Right atrial width	24.85	2.47	24.59	22.45	27.78
Left atrial width	20.43	1.49	20.68	18.46	21.92
Right ventricular width	19.98	1.18	19.49	19.21	21.74
Left ventricular width	30.12	2.70	29.62	27.67	33.57

*Note:* Values are expressed in millimetres.

Abbreviation: SD, standard deviation.

Haematoxylin‐and‐eosin sections showed an epicardium covering the external myocardial surface, an endocardium lining the chamber lumina and myocardium as the predominant component of the chamber walls. Longitudinal cardiomyocytes were elongated and generally contained one or two central nuclei, whereas transverse profiles varied in size among chambers (Figure 3). Silver impregnation demonstrated an argyrophilic reticular network surrounding myocardial fibres in all four chambers (Figure 4). The parietal pericardium comprised a fibrous layer of dense collagenous connective tissue and a thin surface lining compatible with its serous component, although the latter was not consistently preserved. Masson's trichrome clearly demonstrated collagen bundles within the pericardial fibrous layer and collagen within the ventricular myocardial stroma. Collagen‐positive area was greater in the right than in the left ventricle (9.67% ± 1.35% vs. 2.28% ± 0.56% of tissue area; *p* < 0.001; Figure 5).

**FIGURE 3:**
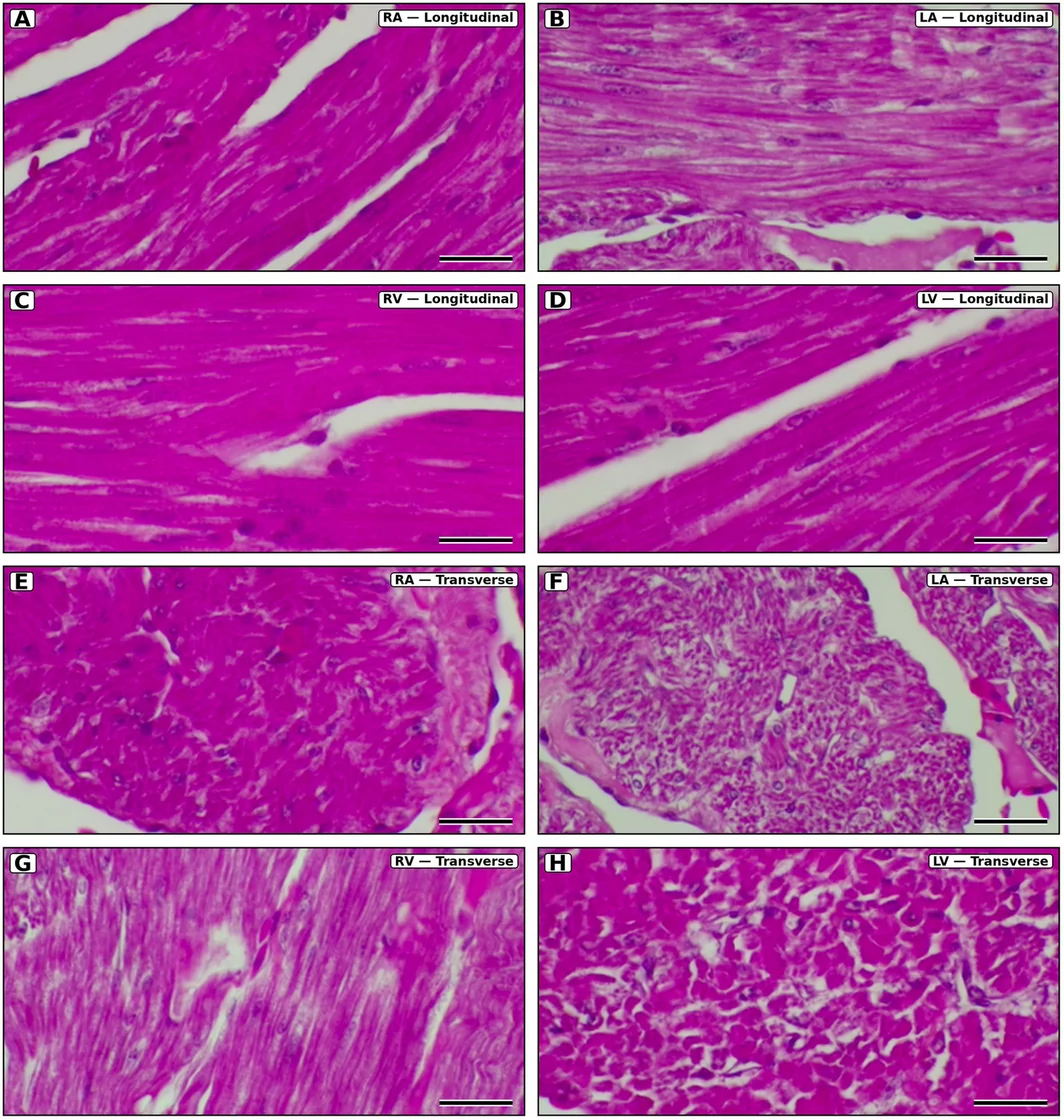
Representative haematoxylin‐and‐eosin sections of the cardiac chambers of *Caiman latirostris* . Longitudinal sections of the right atrium (A), left atrium (B), right ventricle (C), and left ventricle (D), and transverse sections of the right atrium (E), left atrium (F), right ventricle (G), and left ventricle (H). Scale bars = 50 μm.

**FIGURE 4:**
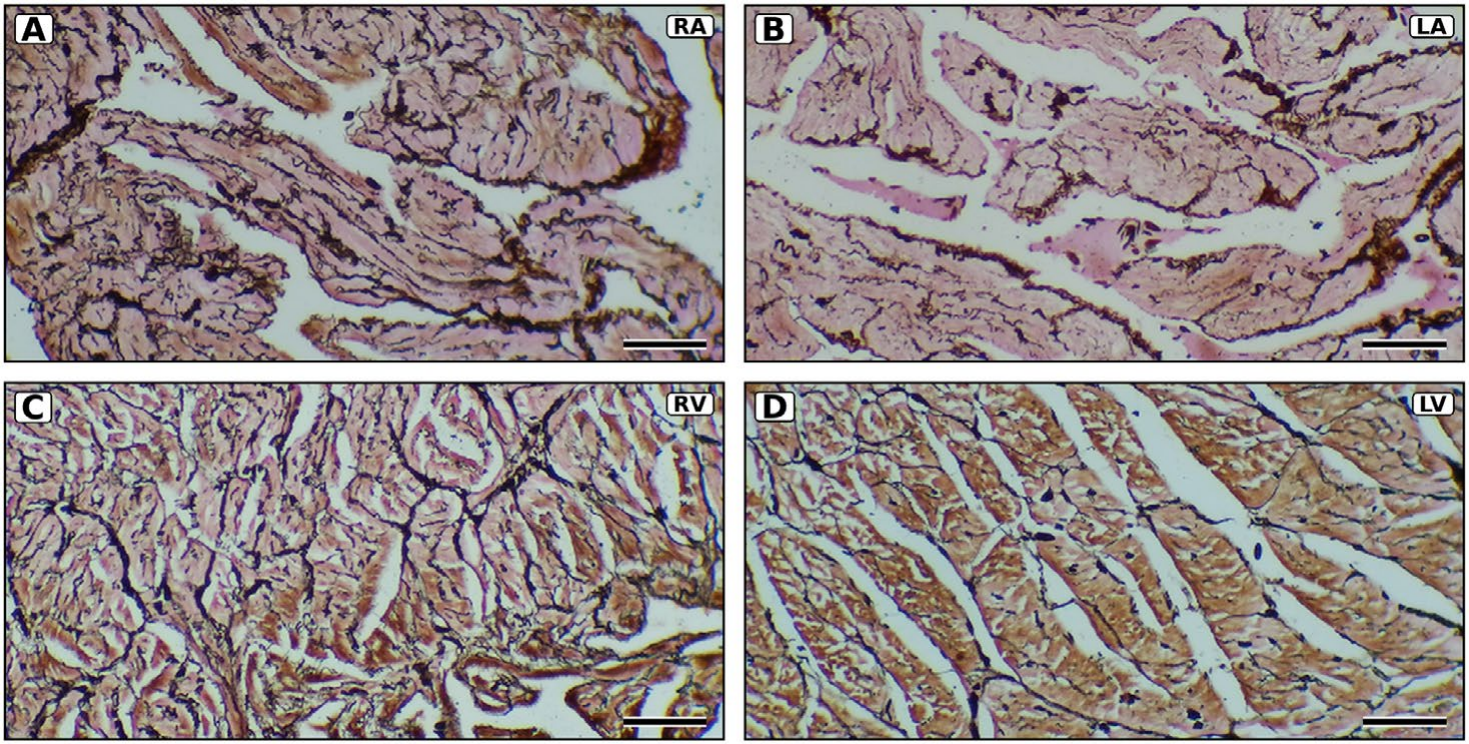
Reticulin‐stained myocardium of *Caiman latirostris* . (A) Right atrium; (B) left atrium; (C) right ventricle; and (D) left ventricle. Argyrophilic reticular fibres appear black and surround the myocardial fibres. Scale bar = 50 μm.

**FIGURE 5:**
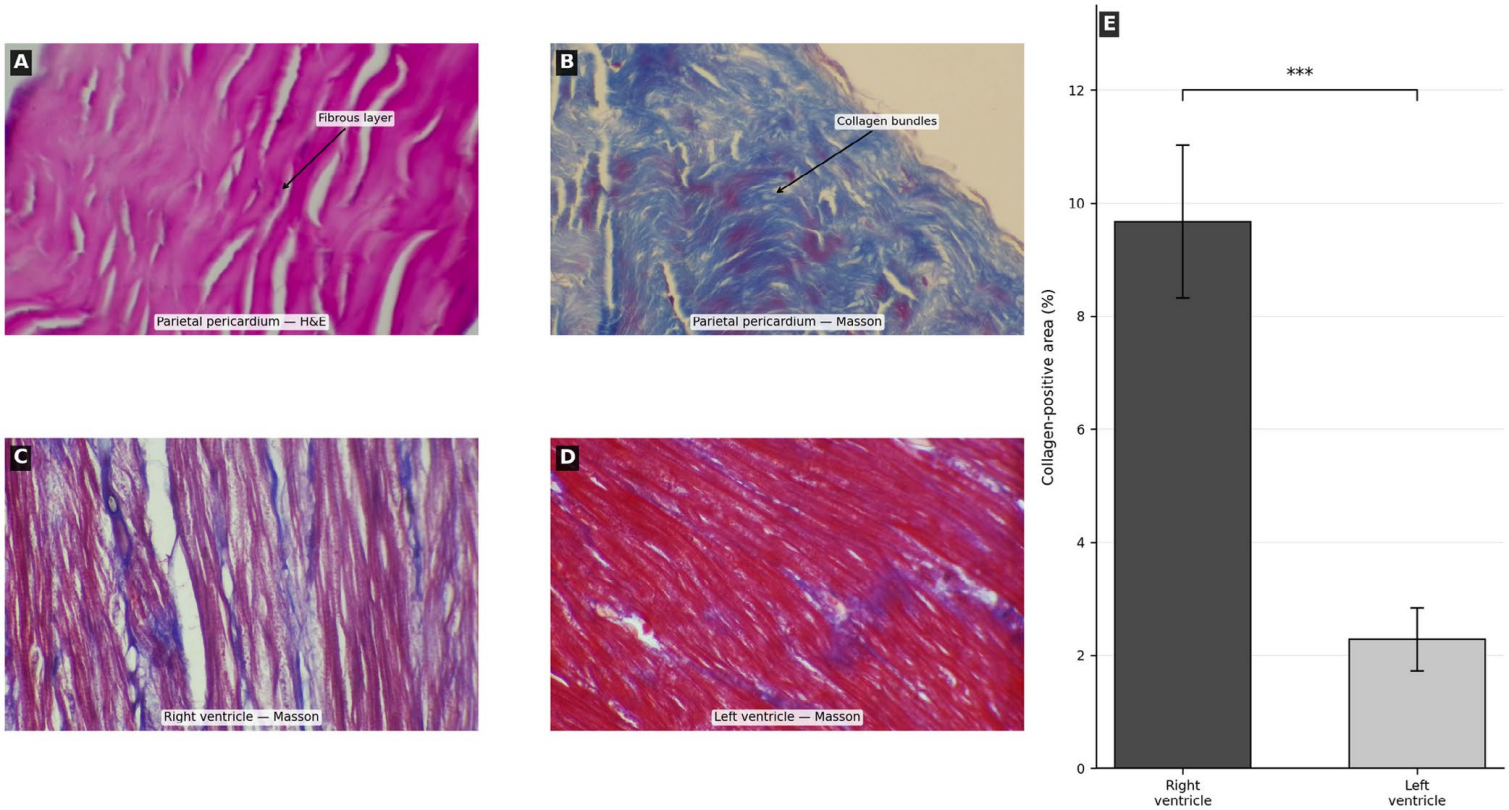
Pericardial histology and ventricular collagen distribution in *Caiman latirostris* . (A) Parietal pericardium stained with haematoxylin and eosin, showing the collagenous fibrous layer; (B) parietal pericardium stained with Masson's trichrome, highlighting collagen bundles in blue; (C, D) right and left ventricular myocardium, respectively, stained with Masson's trichrome; and (E) ventricular collagen‐positive area. Five microscopic fields were averaged per ventricle in each animal. Bars represent the mean and error bars show the standard deviation among four animals; ***p < 0.001.

Cardiomyocyte Vv was greater in the right than in the left atrium and greater in the left than in the right ventricle. Cardiomyocyte cross‐sectional area did not differ between atria but was greater in the left than in the right ventricle (Figure 6). The paired systemic aortae had similar wall thicknesses. Mean wall thickness was 189.44 ± 39.19 μm in the right pulmonary artery and 252.42 ± 64.21 μm in the left pulmonary artery; the left pulmonary arterial wall was thicker (*p* < 0.05; Figure 7).

**FIGURE 6:**
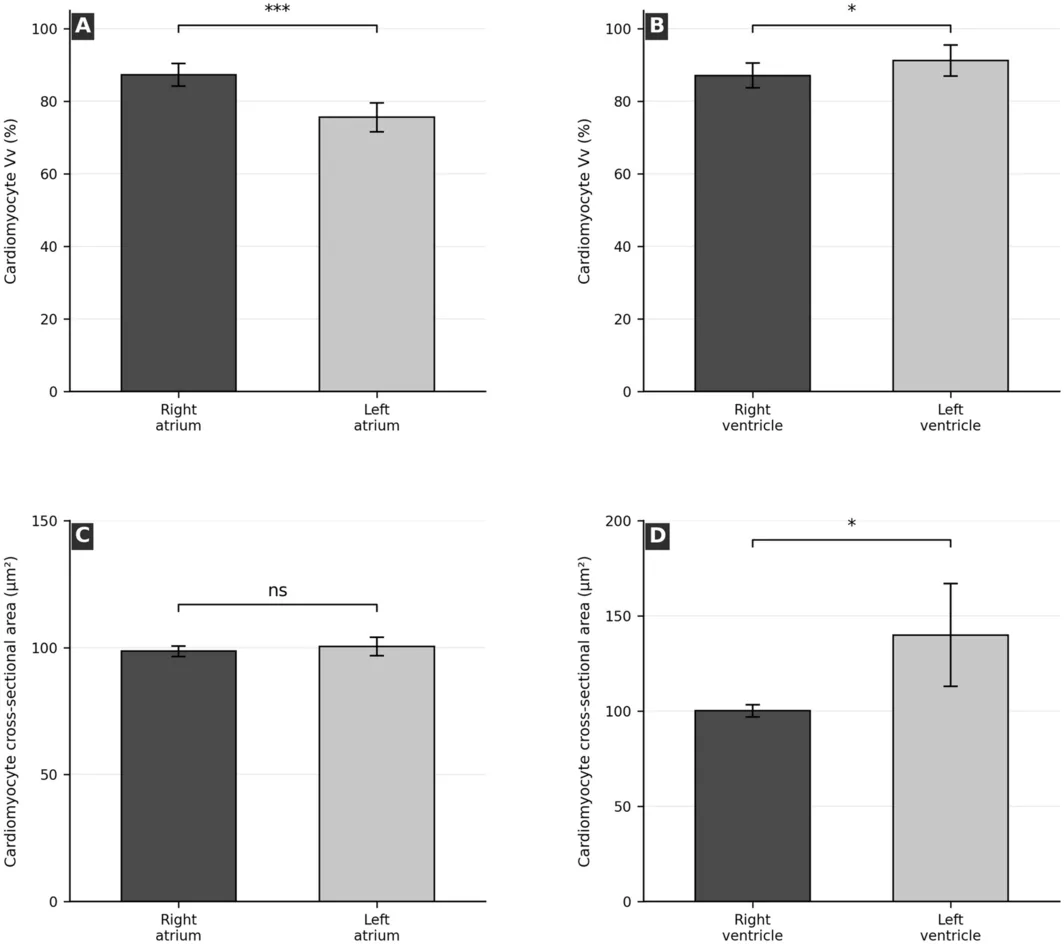
Chamber‐specific cardiomyocyte morphometry in *Caiman latirostris* . Cardiomyocyte volume density (Vv) in the atria (A) and ventricles (B), and cardiomyocyte cross‐sectional area (μm^2^) in the atria (C) and ventricles (D). Data were obtained from four animals, with six microscopic fields averaged per chamber in each animal. Error bars show standard deviation. Atrial Vv: ***p < 0.001; ventricular Vv and ventricular cross‐sectional area: *p < 0.05; atrial cross‐sectional area: *p* > 0.05.

**FIGURE 7:**
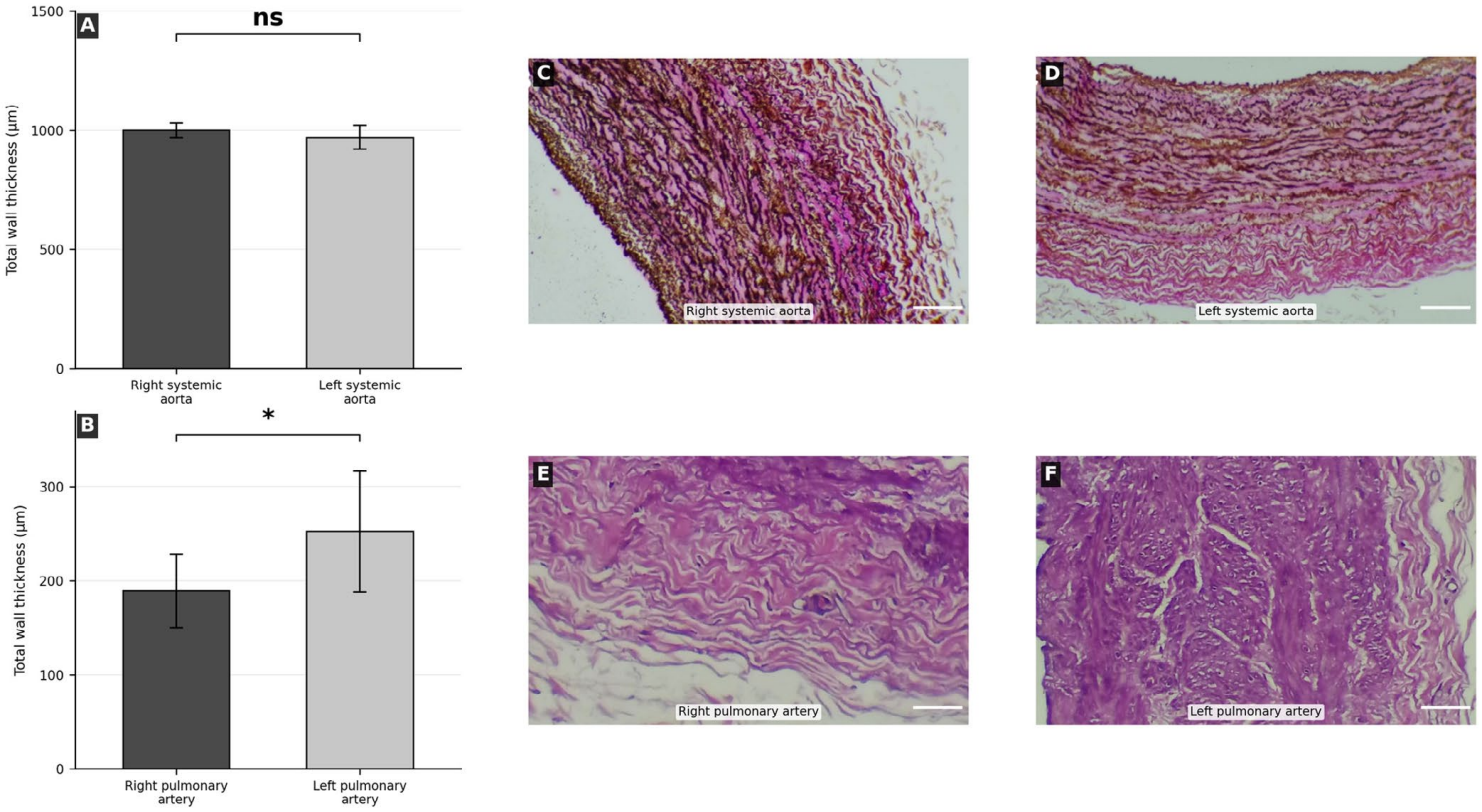
Systemic aortic and pulmonary arterial wall thickness in *Caiman latirostris* . (A) Total wall thickness of the right and left systemic aortae; (B) total wall thickness of the right and left pulmonary arteries; (C, D) representative right and left systemic aortae; and (E, F) representative right and left pulmonary arteries. Three equidistant measurements were obtained per pulmonary artery in each of four hearts. Scale bars = 50 μm in (C, D) and 20 μm in (E, F). Bars and error bars show the mean ± standard deviation. The paired pulmonary comparison showed a thicker left than right arterial wall (*p* < 0.05), whereas the right–left systemic aortic comparison was not significant (*p* > 0.05).

## 4 Discussion

To our knowledge, this is the first study to integrate gross cardiac measurements with chamber‐specific histomorphometry, stereology, ventricular collagen quantification and comparative vascular wall measurements in commercially reared male *C. latirostris* . The anatomical organisation corresponded to the established crocodilian pattern and agreed with descriptions in the same species and other alligatorids (Alves et al. 2016; de Oliveira Lima et al. 2021; Xie et al. 2023). Within this conserved arrangement, the present quantitative findings demonstrate that right–left asymmetry extends beyond chamber dimensions to the cellular and extracellular composition of the myocardium and to the pulmonary arterial walls.

Gross dimensions were broadly consistent with the previous description of *C. latirostris* , as the mean base‐to‐apex length observed here (45.84 mm) approached the 49.86 mm reported by de Oliveira Lima et al. (2021), despite the smaller hearts in the present sample. Both studies examined four hearts fixed in 10% formalin for 48 h; however, de Oliveira Lima et al. evaluated three females and one male with heart masses of 32.38–58.98 g (mean 45.03 g), whereas the present study evaluated four males with heart masses of 15.42–26.67 g (mean 20.64 g). Sampling protocols also differed: the former study measured the right ventricular wall at the base and the left ventricular wall at the base and apex, while the present measurements were standardised to the mid‐ventricular free wall. Consequently, comparisons of absolute dimensions should account for specimen size, sex composition, anatomical sampling level, and physiological state at fixation, particularly because cardiac‐cycle phase and chamber filling were not standardised in the present study or reported previously.

Normalising heart mass to body mass provided an additional basis for comparison across animals of different sizes. Relative heart mass averaged 0.125%, below the approximately 0.25% value compiled for crocodilians by Cook et al. (2017). Because the latter estimate represents a broader taxonomic synthesis rather than a directly matched population, the difference cannot be attributed to a single biological factor; species, age, body size, husbandry and tissue preparation may all contribute. The present values therefore provide a species‐ and population‐specific reference for commercially reared male *C. latirostris* weighing 10.8–23.4 kg.

At the tissue level, the ventricular findings were consistent with different structural demands on the two sides of the heart. The left ventricle had greater cardiomyocyte Vv and cross‐sectional area than the right, indicating a larger cellular contribution to the sampled myocardium. In unanaesthetised *Crocodylus porosus* , mean pressure was 6.2 ± 0.4 kPa in the systemic aortae (*n* = 9) and 2.1 ± 0.1 kPa in the pulmonary artery (*n* = 8), while spontaneous changes in right intraventricular pressure generated a maximum right‐ventricular‐to‐pulmonary‐arterial gradient of 3.3 ± 0.5 kPa (*n* = 3; mean ± SEM; Axelsson et al. 1997). This haemodynamic context is compatible with the larger left ventricular cardiomyocytes and the numerically thicker left ventricular wall observed here, although the wall‐thickness difference did not reach statistical significance. In the atria, greater right‐sided Vv without a corresponding difference in cardiomyocyte cross‐sectional area suggests that tissue occupancy, rather than individual cell size, accounted for the observed asymmetry.

The extracellular matrix showed a complementary right–left pattern. Reticulin staining demonstrated the fine stromal framework surrounding myocardial fibres, whereas Masson's trichrome permitted quantitative assessment of collagen‐positive area. The right ventricle combined lower cardiomyocyte Vv with greater collagen‐positive area, while the left ventricle showed greater cardiomyocyte Vv and cell size. A comparable directional difference has been reported in structurally normal adult human hearts, in which collagen occupied 15.2% of the right ventricular myocardium and 9.5% of the left (Miles et al. 2020). Direct numerical comparison is limited by differences in species, tissue sampling, staining, and analytical methods (Schipke et al. 2017), but the shared direction supports chamber‐specific organisation of the myocardial extracellular matrix.

Vascular wall measurements further reflected the distinction between systemic and pulmonary outflow. The paired systemic aortae had similar wall thicknesses despite arising from different ventricles, possibly because their communication through the foramen of Panizza and shared participation in the systemic circulation expose them to related haemodynamic conditions (Jones and Shelton 1993; Axelsson and Franklin 2001; Cook et al. 2017). Both pulmonary arterial walls were markedly thinner than the systemic aortae, consistent with the lower pulmonary pressure recorded physiologically in crocodilians (Axelsson et al. 1997). The left pulmonary arterial wall was thicker than the right in the present sample; however, the biological basis of this asymmetry remains uncertain and requires confirmation in a larger series.

The study was limited by the small sample of four males from a single closed‐cycle farming system. Cardiac‐cycle phase and chamber filling at fixation were not standardised, and separate chamber weights were unavailable. The irregular compact–trabecular interface in the preserved and dissected specimens precluded reproducible independent measurements of these myocardial components; therefore, only total ventricular wall thickness was quantified. Atrial length and cross‐sectional area were not calculated because the dissections did not provide a consistent plane equivalent to a standard four‐chamber view. Although three equidistant measurements were averaged per pulmonary artery in each animal, the small sample restricts population‐level inference. Future studies combining standardised fixation and filling conditions with three‐dimensional imaging, such as micro‐computed tomography, should refine chamber geometry and test the observed pulmonary arterial asymmetry.

In conclusion, cardiac asymmetry in *C. latirostris* involves gross chamber dimensions, cardiomyocyte organisation, and extracellular matrix distribution. The left ventricular wall was numerically thicker than the right, although the difference was not statistically significant, whereas collagen‐positive area was greater in the right ventricle. The paired systemic aortae had similar wall thicknesses, whereas the pulmonary arterial walls were markedly thinner and the left pulmonary arterial wall was thicker than the right. Together with relative heart mass and chamber‐specific cardiomyocyte measurements, these findings expand the quantitative morphological reference for the broad‐snouted caiman.

## Author Contributions

Conceptualisation: Marcela Aldrovani Rodrigues. Data curation: Maria Eduarda Natal Nascimento and Gabriela Nogueira Maggio. Formal analysis: Marcela Aldrovani Rodrigues. Funding acquisition: Marcela Aldrovani Rodrigues. Investigation: Maria Eduarda Natal Nascimento, Gabriela Nogueira Maggio, Vinícius José da Silva Cardoso de Brito, Arianne Pontes Oriá, Fernanda Gosuen Gonçalves Dias and Marcela Aldrovani Rodrigues. Methodology: Maria Eduarda Natal Nascimento, Gabriela Nogueira Maggio, Maysa Barbosa de Almeida, Caio Rafael Siqueira Vasconcelos, Vinícius José da Silva Cardoso de Brito, Arianne Pontes Oriá, Fernanda Gosuen Gonçalves Dias and Marcela Aldrovani Rodrigues. Writing – original draft: Maria Eduarda Natal Nascimento. Writing – review and editing: Marcela Aldrovani Rodrigues.

## Funding

This Study Was Supported by the Conselho Nacional de Desenvolvimento Científico e Tecnológico (CNPq; Grant 312524/2021‐8) and the Coordenação de Aperfeiçoamento de Pessoal de Nível Superior (CAPES; Finance Code 001).

## Ethics Statement

This study used tissues collected after routine commercial slaughter for meat and leather; slaughter was unrelated to the research, and no experimental procedure was performed on live animals. The farming facility was registered with IBAMA (no. 546538), and specimen collection was authorised by SISBIO/ICMBio (no. 27489–8).

## Conflicts of Interest

The authors declare no conflicts of interest.

## Data Availability

The data supporting the findings of this study are available from the corresponding author upon reasonable request.
